# Graphene sandwich–based biological specimen preparation for cryo-EM analysis

**DOI:** 10.1073/pnas.2309384121

**Published:** 2024-01-22

**Authors:** Jie Xu, Xiaoyin Gao, Liming Zheng, Xia Jia, Kui Xu, Yuwei Ma, Xiaoding Wei, Nan Liu, Hailin Peng, Hong-Wei Wang

**Affiliations:** ^a^Ministry of Education Key Laboratory of Protein Sciences, Beijing Frontier Research Center for Biological Structure, Beijing Advanced Innovation Center for Structural Biology, School of Life Sciences, Tsinghua University, Beijing 100084, China; ^b^Tsinghua-Peking Center for Life Sciences, School of Life Sciences, Tsinghua University, Beijing 100084, China; ^c^Beijing National Laboratory for Molecular Sciences, College of Chemistry and Molecular Engineering, Peking University, Beijing 100871, China; ^d^State Key Laboratory for Turbulence and Complex System, Department of Mechanics and Engineering Science, College of Engineering, Peking University, Beijing 100871, China; ^e^Beijing Graphene Institute, Beijing 100095, China; ^f^Academy for Advanced Interdisciplinary Studies, Peking University, Beijing 100871, China

**Keywords:** cryo-EM, graphene sandwich, sample preparation, beam-induced motion, charging effect

## Abstract

High-quality specimen preparation plays a crucial role in cryo-electron microscopy (cryo-EM) structural analysis. In this study, we have developed a reliable and convenient technique called the graphene sandwich method for preparing cryo-EM specimens. This method involves using two layers of graphene films that enclose macromolecules on both sides, allowing for an appropriate ice thickness for cryo-EM analysis. The graphene sandwich helps to mitigate beam-induced charging effect and reduce particle motion compared to specimens prepared using the traditional method with graphene support on only one side, therefore improving the cryo-EM data quality. These advancements may open new opportunities to expand the use of graphene in the field of biological electron microscopy.

## Significance

Although cryo-EM has become a widely used technique for analyzing high-resolution structures of macromolecules, it still faces several challenges, including significant background noise, beam-induced particle motion, charging effect, and the air–water interface. In this study, we have introduced a graphene sandwich technique that utilizes graphene membranes as both the coverslip and slide to enclose macromolecules for cryo-EM specimen preparation. Due to the superior properties of graphene, such as low background noise and strong mechanical strength, this graphene sandwich-based cryo-EM specimen preparation method has demonstrated an improvement in the quality of collected cryo-EM datasets when compared to the conventional approach of using graphene support on just one side.

Cryogenic electron microscopy (cryo-EM) has become a powerful tool to determine the native and high-resolution architectures of sensitive materials in the fields of structural biology (1) and material sciences (2). Cryo-EM analysis involves freezing hydrated samples and characterizing them at liquid nitrogen temperature (3, 4). Recent algorithm and hardware breakthroughs have boosted the technique’s structure-solving efficiency (5, 6) and enabled structural determination at genuine atomic resolution for macromolecules (7, 8). However, high-quality specimen preparation still faces many critical challenges (9, 10), becoming one of the major rate-limiting steps in cryo-EM structure determination. Problems such as the air–water interface (AWI), strong background noise, preferential orientation, beam-induced particle motion, and radiation damage frequently impair the final high-resolution structural reconstruction (11, 12).

Graphene, a two-dimensional material that is only one atom thick, exhibits superior electrical conductivity (13, 14) and mechanical strength (15). Graphene and its derivatives have been developed as supporting films for cryo-EM specimen preparation, offering advantages such as improved particle adsorption and reduced beam-induced particle motion (16–20). Bioactively functionalized graphene film can effectively modulate interactions with target particles and alleviate the AWI perturbation (21–25). When using graphene films for cryo-EM specimen preparation, the process begins by incubating the sample solution with the graphene support. Next, the solution is blotted with filter papers to create a thin layer of aqueous film. This film is then rapidly frozen by plunging it into liquid ethane, which has been pre-cooled by liquid nitrogen. As a result, a thin vitreous ice layer forms on the graphene support, containing the target molecules. However, in this cryo-specimen, the graphene support is only present on one side, leaving the other side exposed and thereby still posing a potential risk of AWI perturbation. While sandwich structures using amorphous carbon film (26) or silicon-rich nitride film (27) as both the slide and coverslip for cryo-EM specimen preparation have been developed, these films exhibit poorer electrical conductivity and generate significant background noise. Additionally, the procedure for fabricating the silicon-rich nitride sandwich chip is technically challenging and expensive.

Previous studies have explored the use of graphene sandwich technique to encapsulate sample solutions on both sides, enabling the fabrication of graphene liquid cells (GLCs) for room-temperature TEM analysis, also known as liquid-phase TEM (28). The GLCs are formed between the two layers of graphene due to the impermeability of graphene membranes to molecules like water and gases (29, 30), and the van der Waals forces between the graphene layers effectively seal the sample solution. Within the GLCs, the enclosed objects remain hydrated and can freely diffuse (31), providing an ideal platform for investigating in situ crystal growth (32) and radical behavior (33). However, the procedures previously reported for using a graphene sandwich to produce GLCs were fragile, susceptible to polymer contaminations, and had a high risk of graphene curling and cracking. As a result, the encapsulation success rates were unreliable, and the reproducibility of GLC fabrication was limited (34).

**Fig. 1.**
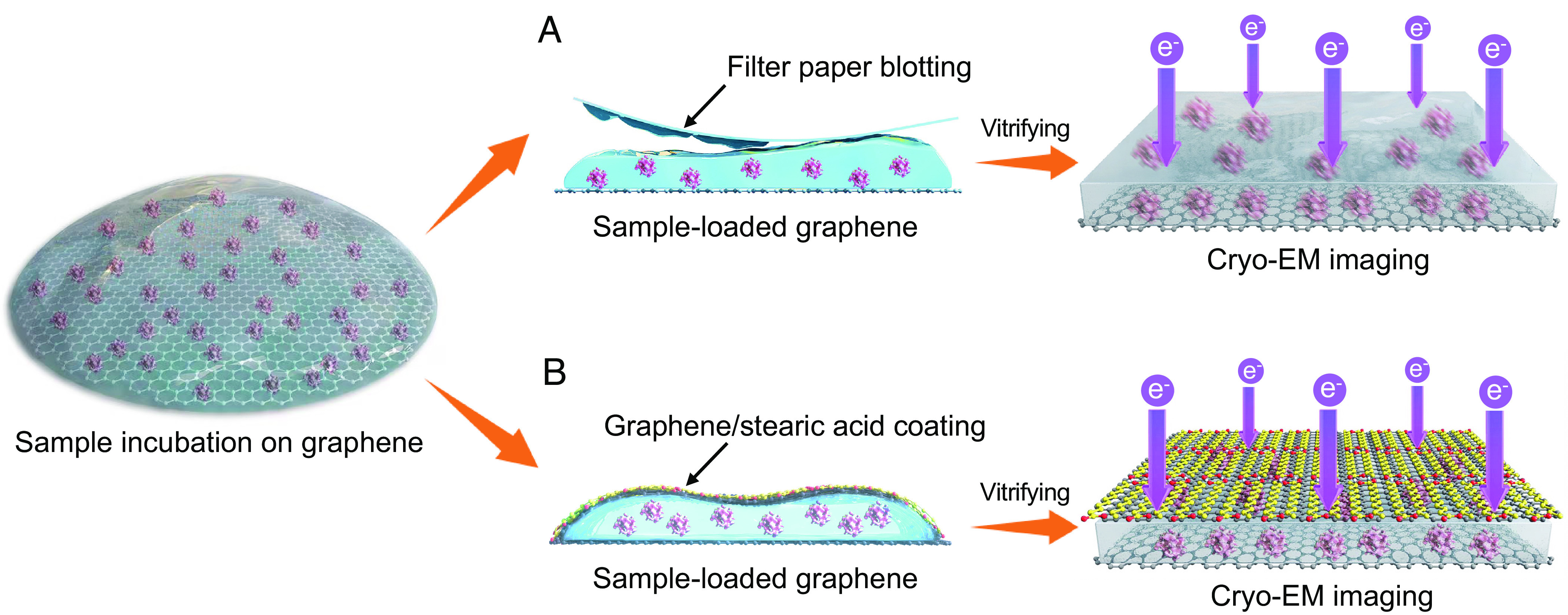
Schematic illustration showing cryo-EM specimen preparation using graphene as the supporting film. (*A*) The conventional specimen preparation method that involves incubating the sample solution on a graphene grid and subsequently blotting it with filter paper prior to plunge freezing for cryo-EM analysis. (*B*) Specimen preparation by the graphene sandwich approach, in which an additional layer of graphene film with stearic acid coating is placed onto the sample-loaded graphene grid, forming an aqueous film before vitrification for cryo-EM analysis.

In this study, we have developed a robust approach to fabricating the graphene sandwich by transferring a second layer of free-standing and flat graphene film onto a graphene grid that holds a droplet of sample solution (Fig. 1). This process generates a thin aqueous film, securely enclosed between the two layers of graphene films (Fig. 1*B*). Compared to the conventional specimen preparation method using a single-sided graphene film (Fig. 1*A*), the graphene sandwich improves the cryo-EM data quality by reducing the charging effect and beam-induced particle motion. Using this method, we successfully determined the high-resolution cryo-EM structures of 20S proteasome, apoferritin, and SARS-CoV-2 spike protein.

## Results

### Fabrication and Characterization of the Graphene Sandwich.

**Fig. 2.**
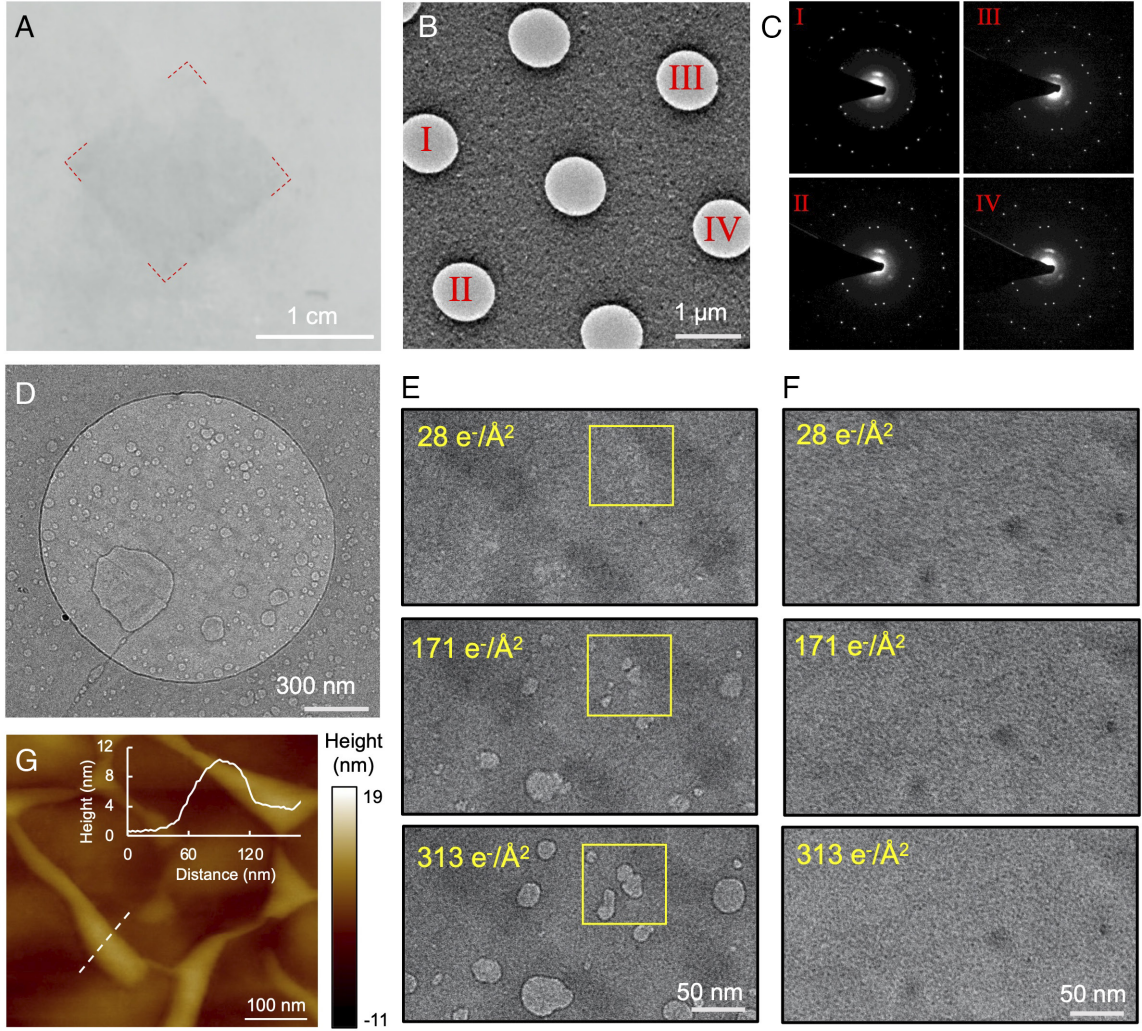
The characterization of graphene sandwiched specimen. (*A*) Free-standing graphene film on the surface of the solution. (*B*) A representative room-temperature TEM micrograph of the graphene sandwiched specimen. (*C*) Selected area electron diffraction (SAED) images of the labeled regions in *B* demonstrating two layers of graphene. (*D*) A representative room-temperature TEM image of graphene sandwiched aqueous specimen after electron beam irradiation, revealing a large number of bubbles due to radiation damage of the solution. (*E*) Room-temperature TEM characterization of the bubble behaviors in graphene sandwiched specimen with accumulated dose. (*F*) Room-temperature TEM characterization of one-side graphene support with accumulated dose. (*G*) A representative AFM image of the graphene sandwiched specimen. The *Inset* showed the height profile across the dotted white line.

To fabricate the graphene sandwich, we first prepared a free-standing graphene film on an aqueous surface (Fig. 2*A*). Specifically, we used graphene films that were synthesized by the chemical vapor deposition (CVD) method (35). The air-facing side of graphene was coated with stearic acid molecules by a procedure described further in the *Materials and Methods* section. These molecules self-assembled into thin-layer crystals on the graphene surface, helping to preserve the graphene’s integrity during the subsequent etching step (*SI Appendix*, Fig. S1). After etching off the copper foil, the graphene film remained free-standing and intact, making it easy to handle and robust for transfer onto any desired substrate. Following that, we transferred the free-standing graphene film onto a graphene grid with sample solution using either the direct-scooping method or the loop-assisted method (*SI Appendix*, Fig. S2). Finally, we placed the grid carrying the graphene sandwich onto a piece of filter paper to remove the overflow and suck away the excess solution from the sandwiched area.

We conducted room-temperature TEM characterization of the graphene sandwich suspended on the EM grid and observed no visible contaminations (Fig. 2*B*). SAED displayed two sets of diffraction spots with hexagonal symmetry, which were consistently oriented across the observed holes (Fig. 2*C*). This observation suggests that the graphene sandwich depicted in Fig. 2*B* was likely formed by stacking two single-crystal graphene layers. Under low-magnification TEM, the graphene sandwich exhibited unevenly distributed contrast within the holes (*SI Appendix*, Fig. S3*A*). The regions with darker contrast corresponded to areas where the cells contained a higher amount of aqueous solution. Following high-dose irradiation, multiple bubbles appeared in the illuminated region (Fig. 2*D* and *SI Appendix*, Fig. S3*D*), consistent with the previous observations of GLCs (36, 37). These bubbles were generated due to the electron bombardment of water molecules, leading to the decomposition of these molecules and the production of gas and radicals, which were encapsulated within the graphene sandwich. As a control, we conducted room-temperature TEM analysis on the graphene film coated with stearic acid by transferring it to an EM grid covered with holey carbon film (*SI Appendix*, Fig. S4). This analysis showed no formation of bubbles even under high-dose electron illumination. These phenomena confirmed the presence of a liquid phase within the graphene-sandwiched area and demonstrated that the graphene sandwich effectively prevented liquid evaporation within an ultrahigh-vacuum microscope chamber.

To investigate the relationship between electron dose of irradiation and bubble formation, we collected a series of micrographs on the same area with consecutive exposures (Fig. 2*E* and *SI Appendix*, Fig. S5). We observed that as the dose increased, the bubbles tended to grow larger in size, and some instances of bubble fusion indicated gas diffusion within the graphene sandwich. As a control, we prepared specimens with graphene support on one side only, where the sample solution was added to the graphene grid without covering the second layer of graphene film. In this case, no irradiation-induced bubbles were observed in the specimen, even after illumination with an accumulated dose of 313 e^−^/Å^2^ (Fig. 2*F*). This outcome was expected since the aqueous solution was not sealed within the single-sided graphene film, causing rapid evaporation under the high-vacuum conditions of TEM. We also conducted electron illumination on two stacked graphene layers, which were created by transferring one layer of graphene onto a graphene grid, and we did not observe any visible bubbles (*SI Appendix*, Fig. S6). Atomic force microscopy (AFM) analysis revealed the presence of graphene liquid cells of varying sizes on the graphene-sandwiched specimen (Fig. 2*G*), confirming successful encapsulation. Collectively, our method of fabricating the graphene sandwich successfully encapsulated the target sample solutions, demonstrating its suitability for preserving hydrated samples for electron microscopy analysis.

### Graphene-sandwiched Specimen for High-resolution Cryo-EM Structure Determination.

**Fig. 3.**
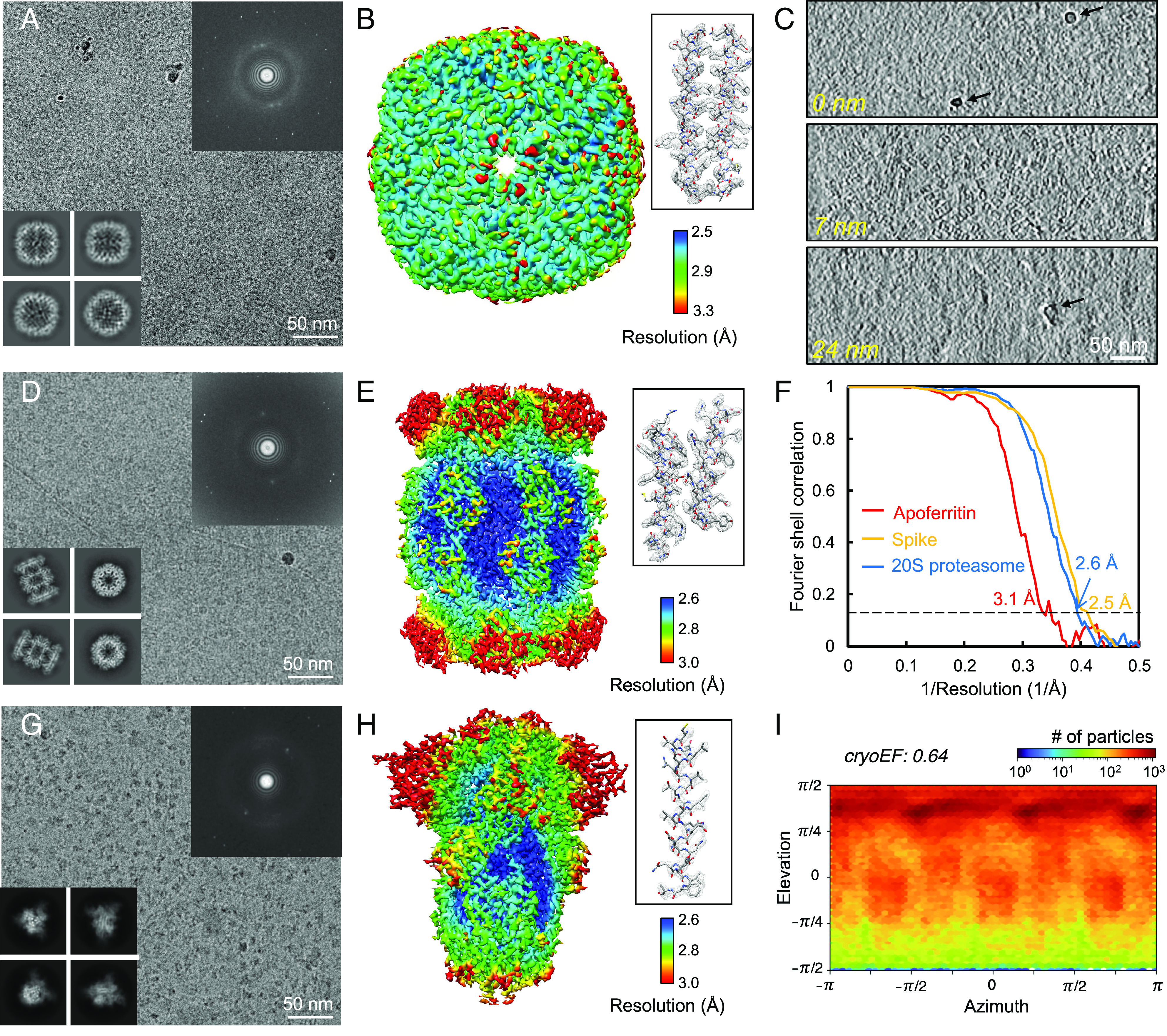
Graphene sandwich for cryo-EM structure determination. (*A*) A representative cryo-EM micrograph of graphene-sandwiched apoferritin specimen. The *Bottom-Left Inset* displays the representative two-dimensional (2D) class averages. The *Upper-Right Inset* exhibits the Fourier transform of the image, where the hexagonally symmetric diffraction spots are attributed to multilayer graphene and the spots closer to the central beam correspond to the stearic acid lattice. (*B*) The three-dimensional (3D) cryo-EM density of apoferritin (EMD-37156), reconstructed using the particles from micrographs like (*A*). The surface of the density was colored according to the local resolution distribution. The *Inset* shows a partial density with the corresponding atomic model docked. (*C*) Selected slices from the cryo-ET reconstruction of graphene-sandwiched 20S proteasome specimen. The top slice corresponds to the upper boundary, referred to as the 0-nm layer. The middle slice corresponds to the layer in the ice where the particles are distributed, located 7 nm away from the upper boundary. The bottom slice corresponds to the lower boundary, 24 nm away from the upper boundary. Some ice contaminations at the lower and upper boundaries are labeled with black arrows. (*D*) A representative cryo-EM image of graphene-sandwiched 20S proteasome specimen. The *Bottom-Left Inset* displays the representative 2D class averages. The *Upper-Right Inset* is the Fourier transform of the image. (*E*) The 3D cryo-EM structure of 20S proteasome (EMD-37158), reconstructed using the particles from micrographs like (*D*). The *Inset* shows a partial density with the corresponding atomic model docked. (*F*) The plot of Fourier shell correlation (FSC) of the reconstructions of 20S proteasome (blue), apoferritin (red), and spike (orange), respectively. The dotted line indicates FSC = 0.143, used for estimating the resolution. (*G*) A representative cryo-EM micrograph of graphene-sandwiched spike specimen. (*H*) The 3D cryo-EM density of spike (EMD-38213), reconstructed using the particles from micrographs like (*G*). (*I*) The Euler angular distribution of graphene-sandwiched spike protein. The efficiency of orientational distribution (cryoEF value) (38) is also indicated.

We then utilized the graphene sandwich technique to prepare cryo-EM specimens of apoferritin, 20S proteasome and SARS-CoV-2 spike protein, and subsequently collected the corresponding datasets (Fig. 3 and *SI Appendix*, Fig. S7). Upon inspecting the raw micrographs, we observed clear individual particles for all three samples. Furthermore, the Fourier transform of the micrographs exhibited multiple sets of hexagonal diffraction spots, which originated from the graphene films (Fig. 3 *A*, *D*, and *G*). Interestingly, we also detected additional diffraction spots in the Fourier transforms, appearing at half the spatial frequency of the graphene lattice diffraction. These spots were attributed to the stearic acid layer assembled on the surface of the graphene.

Cryo-electron tomography (cryo-ET) analysis of the graphene-sandwiched specimens revealed that the 20S proteasome particles were predominantly distributed in the same plane (*Middle* panel in Fig. 3*C*), with distances of 7 nm and 17 nm from the upper and lower boundaries, respectively. The ice thickness was therefore approximately 24 nm, which was suitable for embedding 20S proteasome particles. Based on the volume of tomogram (Fig. 3*C*), we calculated the 20S proteasome particle concentration within the ice to be approximately 87 μM. Consequently, the particle concentration in the graphene-sandwiched ice was significantly higher than that in the bulk solution before being applied to the grid, amounting to about 0.57 μM, representing an increase of over two orders of magnitude. This enrichment in particle concentration may be attributed to the adsorption of particles onto graphene surface before the formation of graphene sandwich.

We further analyzed the ice thickness of 193 holes using a method that compares the intensity variation at specific locations with and without an energy filter (39). Our results indicated that the average ice thickness in the data collection area on the graphene-sandwiched grid was approximately 60 nm (*SI Appendix*, Fig. S10).

Following a standard data-processing workflow (*SI Appendix*, Fig. S8), we successfully determined the cryo-EM structures of apoferritin, 20S proteasome, and spike protein at resolutions of 3.1 Å, 2.6 Å, and 2.5 Å, using particle images from 55, 50, and 2,200 micrographs, respectively (Fig. 3 and *SI Appendix*, Table S1). In the 3D reconstruction of graphene-sandwiched spike protein, which has less symmetry and stability compared to apoferritin and proteasome, 35.4% of the initially picked particles from the raw micrographs were kept for the final reconstruction (*SI Appendix*, Table S1), a much higher proportion compared to that on the holey carbon film, which ranged from 10.2 to 23.8% (40–43). These results collectively demonstrate the graphene sandwich method's ability to promote the cryo-EM dataset quality and determine high-resolution cryo-EM structures of various macromolecules.

### Characterization of Cryo-EM Data Quality of the Graphene-sandwiched Specimen.

We further examined the data quality of the graphene-sandwiched cryo-specimen, focusing on key challenges that have impeded high-resolution cryo-EM structural determination, including the beam-induced charging effect, particle motion, and radiation damage.

**Fig. 4.**
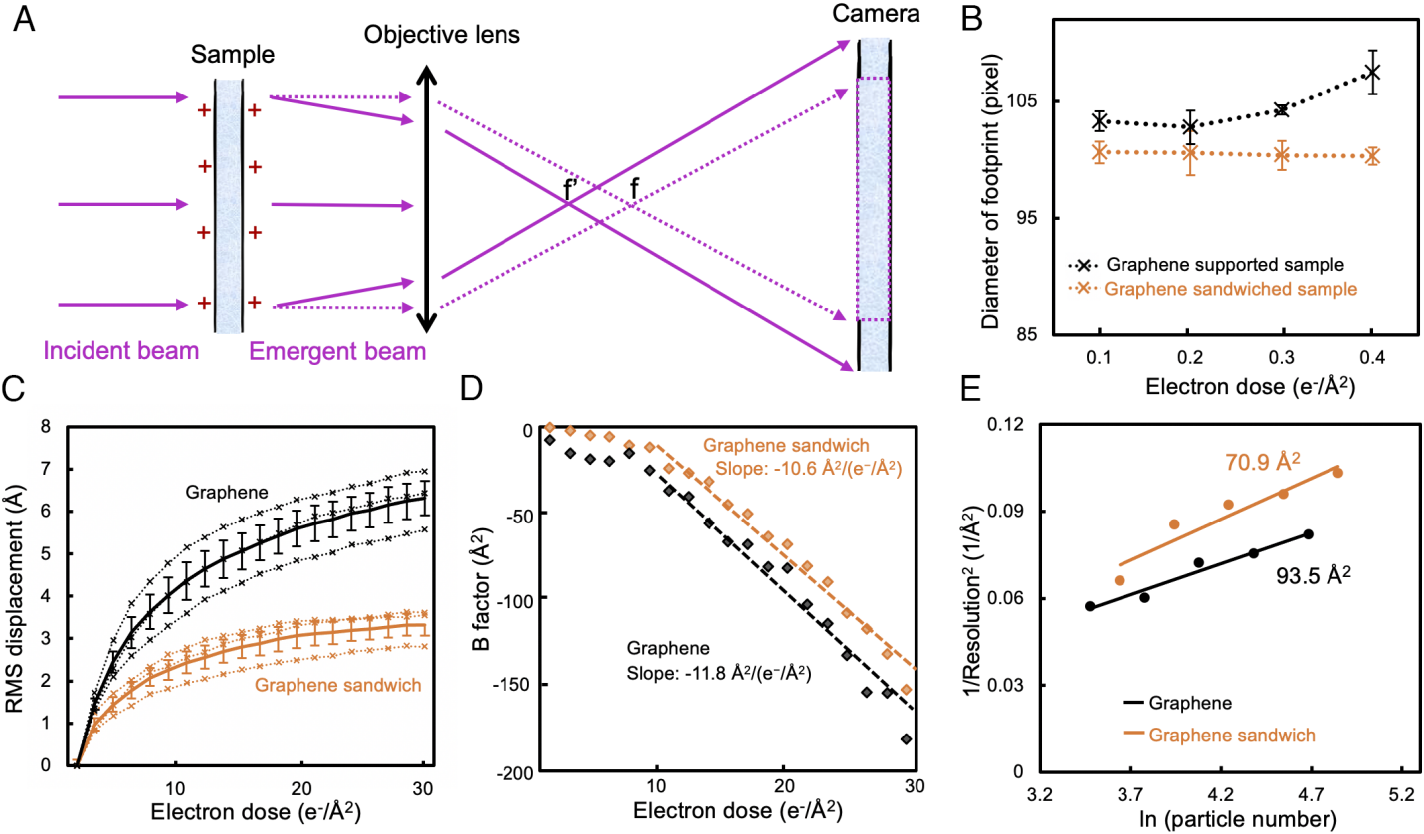
Cryo-EM characterization of the graphene sandwich. (*A*) Illustration showing the changes in the optical path caused by the charging effect. The dashed line represents the imaging optical path without the charging effect perturbation, while the solid line represents the actual imaging optical path. The charging effect causes the emergent beam to converge, consequently shifting the effective focal point toward the sample. (*B*) The diameters of the charging footprints for the graphene-sandwiched sample (orange) and the graphene-supported sample (black) were plotted as a function of the irradiation dose. (*C*) Beam-induced particle displacement with the accumulated dose in the graphene-sandwiched sample (orange) and the graphene-supported sample (black). The distance between particles in the given frame (electron dose) and the first frame was taken as the particle displacement. Every dotted lines represent the average displacement of thousands of particles, and the solid lines represent the average of the dotted lines. (*D*) The per-frame B factor plots of the graphene-sandwiched sample (orange) and the graphene-supported sample (black) with the accumulated electron dose. The slopes of the B factor decrease of the graphene-sandwiched sample (orange) and the graphene-supported sample (black) were −10.6 Å^2^/(e^−^/Å^2^) and −11.8 Å^2^/(e^−^/Å^2^), respectively. (*E*) The overall B factor plots of graphene-sandwiched apoferritin (solid orange line) and graphene-supported apoferritin (solid black line), respectively.

The charging effect is a common problem encountered during cryo-EM data collection, which can perturb the imaging parameters. When a region is irradiated and emits secondary electrons without receiving compensating electrons from the surrounding area, it becomes positively charged. The build-up of positive electrostatic potential acts as a convergent lens (Fig. 4*A*), generating a noticeable footprint in the highly defocused micrograph (*SI Appendix*, Fig. S11). As illustrated in Fig. 4*A*, an increase in the positive charge of the charging region leads to a more pronounced convergence of the emerging beam, which leads to a greater magnification effect of the charging region in the micrograph.

Graphene film exhibits significantly higher electrical conductivity compared to amorphous carbon foil and vitreous ice (26, 44), which might help to alleviate the charging effect. To verify this assumption, we calculated the diameter of the charging footprint in the highly defocused micrographs obtained under the same imaging conditions for both the graphene-sandwiched and graphene-supported samples. Our finding revealed that even a low electron dose of 0.1 e^−^/Å^2^ induced a significant charging effect in both samples (Fig. 4*B*). However, the graphene-sandwiched sample displayed a smaller charging footprint size, indicating a reduced magnification effect and suggesting a decrease in charging effect in the illuminated region (Fig. 4*B*). Furthermore, when examining contamination particles located outside but near the illuminated region of the graphene-supported sample (*SI Appendix*, Fig. S11 *A*–*C*), noticeable changes in contrast were observed compared to the graphene-sandwiched sample (*SI Appendix*, Fig. S11 *D*–*F*). These changes in intensity of the contaminations occur because electrons emitted from the primary irradiation region diffuse into the surrounding area, resulting in a negative charge (45). This observation further suggests that the charging effect is alleviated in the graphene-sandwiched specimen.

Particle motion impairs the recording of high-resolution information in cryo-EM, and one of the major causes of beam-induced motion is ice buckling. In the previous report, the ice buckling occurs when the compression of the ice layer exceeds a critical value *N*_0_ (*SI Appendix*, Fig. S12) (12), which is directly proportional to the Young's modulus of ice. By using a graphene film with a significantly higher Young's modulus than ice, it becomes possible to increase *N*_0_ and prevent ice buckling (46). We estimated that the effective Young's modulus of the graphene-sandwiched ice was approximately 21.8 GPa, roughly twice that of the graphene-supported ice at around 11.5 GPa. Subsequently, we examined the particle motion during cryo-EM imaging and observed that the graphene sandwich yielded reduced motion in comparison to the graphene support on one side. Notably, the displacement of particles in the final frame, relative to their initial position, decreased by approximately two-fold (Fig. 4*C*). Moreover, particle motion was effectively reduced by 1.5 to 2-fold in the first five to six frames (*SI Appendix*, Fig. S13), which preserved the most valuable high-resolution information as these frames experienced less radiation damage compared to the subsequent frames.

To assess the extent of radiation damage, we performed the Bayesian polishing in Relion (47), a program to relatively weight damage across various movie frames, to determine the per-frame B factors of the graphene-supported and graphene-sandwiched apoferritin samples. Both cryo-EM datasets were acquired on the same day, under identical conditions. We found that the graphene-sandwiched apoferritin sample exhibited a substantially smaller B factor, indicative of better data quality. However, the rate at which its B factor decayed with the accumulated dose [−10.6 Å^2^/(e^−^/Å^2^)] was similar to that of the graphene-supported sample [−11.8 Å^2^/(e^−^/Å^2^)], suggesting that the use of graphene sandwich for cryo-EM reconstruction provided little or no alleviation of radiation damage. The overall B factor, which relates the particle number and the achieved resolution and is often used to demonstrate the overall quality of collected datasets (48), was significantly smaller for the graphene-sandwiched sample (70.9 Å^2^) compared to the graphene-supported sample (93.5 Å^2^) (Fig. 4*E*), demonstrating the capability of the graphene sandwich to improve the quality of cryo-EM data.

## Discussion

In this work, we have introduced a straightforward approach for creating a graphene sandwich using graphene films as both the slide and coverslip. Our results have shown that this graphene sandwich method can be employed to prepare cryo-EM specimens without the need of filter paper blotting. This allows for the encapsulation of macromolecules between graphene layers, eliminating contact with the air–water interface. By employing this technique, we achieved an appropriate ice thickness for high-resolution structure determination of macromolecules. Additionally, we have demonstrated the use of graphene sandwich for cryo-EM specimen preparation can help to mitigate the charging effect and beam-induced particle motion when compared to the conventional method of using graphene at only one side. This improvement is attributed to the improved electrical conductivity of graphene and its increased effective Young's modulus. Consequently, the quality of the cryo-EM specimen was improved compared to specimens supported by graphene on only one side.

However, we did not observe an obvious reduction in radiation damage when comparing the cryo-EM reconstruction results using particles in the graphene sandwich and those on graphene support. This finding contrasts with a previous study where the addition of graphene was reported to mitigate radiation damage by scavenging beam-induced radicals in GLC (33, 49). One possible explanation for our results is that the graphene film used in our cryo-samples is located at the boundaries of the specimen, and the diffusion of beam-induced radicals at cryogenic state is significantly restricted compared to that in GLC. Consequently, the graphene at the specimen boundaries is unable to interact with the radicals and prevent damage.

Irradiation with an electron dose as low as 0.1 e^−^/Å^2^ was sufficient to generate noticeable charging footprints. Furthermore, in the case of the graphene-sandwiched sample, the size of the charging footprints remained consistent even when the imaging dose was increased by a factor of four (Fig. 4*B*), suggesting that the charging effect reached a saturation point at an electron dose of 0.1 e^−^/Å^2^. Importantly, this dose is much lower than the dose experienced by the first frame (typically over 1 e^−^/Å^2^) in the current strategy for collecting data in single-particle cryo-EM. This implies that the current data collection method cannot avoid the influence of charging effect. However, fortunately, the charging effect causes minimal contrast loss and has almost no effect on beam-induced motion in single-particle cryo-EM since the charging accumulates at an extremely low dose (less than 0.1 e^−^/Å^2^) and then remains constant (50).

In summary, we explored the use of graphene sandwich for cryo-EM sample preparation. This approach led to improved cryo-EM data quality and enabled high-resolution structure determination. We would like to emphasize that in future optimization efforts, modifying the interior surface of the graphene sandwich, that is, the sample-facing graphene surface, in a structure-friendly way would be a better and more universally applicable approach for numerous protein complexes. Additionally, we propose the incorporation of a holey flake with an appropriate thickness into the graphene sandwich to achieve uniform control over the ice thickness, similar to the well-type GLC (51), which employs a patterned spacer between the graphene layers to regulate the volume and thickness of the enclosed solution.

## Materials and Methods

### Production of Free-standing Graphene Film.

Large-scale single-crystal graphene film was first synthesized on copper foil (Kunshan Luzhifa Electron Technology Co., Ltd., China) using the chemical vapor deposition (CVD) method (35). The graphene-coated copper foil could also be purchased directly from the Beijing Graphene Institute (BGI) Co. Ltd. The graphene film on the copper foil backside was removed via plasma treatment in a reactive ion etcher (Pico SLS, Diener) under the atmosphere of airflow (10 sccm) and the power of 150 W for 3 min. Afterward, a small drop of isopropanol (IPA) solution containing 0.005% stearic acid molecules was added onto the untreated side of copper foil, that is, the graphene side, which was then left for air-drying. For a graphene/copper foil with an area of 1.5 cm × 1.5 cm, we added approximately 10 μL of the stearic acid/IPA solution. After the air-drying process, there would be two to three layers of stearic acid molecules on the graphene surface. The resulting graphene/copper foil coated with stearic acid molecules was floated on the surface of 0.5 M (NH_4_)_2_S_2_O_8_ solution for about 1 h to completely etch off the copper foil, and the free-standing graphene film was obtained on the aqueous surface. The free-standing graphene was then gently transferred onto the surface of a water or buffer solution using a piece of stainless-steel mesh (Movie S1), ready to be used for fabricating the graphene sandwich.

### Preparation of Biological Samples.

We purchased ribosomes from New England Biolabs (Catalog: P0763S) and diluted them to 1.11 mg/mL for use. Apoferritin was purchased from Sigma-Aldrich (Catalog: A3660) and diluted to 3.5 mg/mL for use. The 20S proteasome sample was purified from the *Escherichia coli* cells and diluted to 0.4 mg/mL for use. The SARS-CoV-2 spike protein (0.4 mg/mL) was kindly provided by Qiang Zhou at Westlake University.

### Fabrication of Graphene-sandwiched Samples.

To prepare the graphene sandwich, 3 μL of sample solution was pipetted onto a graphene grid, which had been glow-discharged in a plasma cleaner machine (Harrick Plasma) for 15 s at a low power setting. We developed two alternative methods for sandwich preparation: 1) the sample-loaded graphene grid was first immersed beneath the free-standing graphene film and then scooped it up rapidly; 2) the sample-loaded graphene grid was placed on a cylinder stand with a slightly larger diameter than the grid, and the free-standing graphene film was scooped up using a loop, which was then passed through the stand to cover the graphene grid (*SI Appendix*, Fig. S2). Afterward, the grid carrying the graphene sandwich was positioned on a piece of filter paper, which aided in removing any excess solution from the sandwich area. We didn't remove the stearic acid from the upper free-standing graphene surface but instead used it directly for the following EM analysis.

### AFM and Room-temperature TEM Characterization of Graphene Sandwich.

The morphologies of the graphene sandwich on EM grid were characterized by AFM using tapping mode (Bruker Dimension Icon with Nanoscope V controller). Room-temperature TEM characterization was performed using a Tecnai F20 TEM (Thermo Fisher) equipped with a Gatan US4000 (895) CCD in low-dose mode. In the search mode, micrographs were acquired at 1700× magnification to visualize the overall map of the graphene sandwich. In the exposure mode, micrographs were acquired at 29,000× magnification with a pixel size of 3.28 Å, using a dose rate of approximately 57 e^−^/(Å^2^ s) and an exposure time of 0.5 s. To observe the bubble behavior resulting from radiation damage, we captured micrograph series under the above conditions.

### Cryo-EM Analysis of the Graphene-sandwiched Macromolecules.

The aforementioned grid carrying the graphene sandwich was manually inserted into liquid ethane precooled with liquid nitrogen and then transferred to liquid nitrogen for cryo-EM imaging. The single-particle cryo-EM dataset of the graphene-sandwiched and graphene-supported apoferritin was automatically collected using the EPU software on a Thermo Fisher Scientific Titan Krios G3i (300 kV) TEM equipped with a K3 camera (Gatan, Inc.), a GIF-Quantum energy filter (20 eV), and a Cs-corrector. A total of 55 and 100 micrographs were collected with a pixel size of 0.856 Å for the graphene-sandwiched and graphene-supported apoferritin samples, respectively. For the dataset of graphene-sandwiched 20S proteasome, 50 micrographs were collected with a pixel size of 0.8374 Å via AutoEMation software (52) on a Thermo Fisher Scientific Titan Krios (300 kV) TEM equipped with a K3 camera (Gatan, Inc.) and a GIF-Quantum energy filter (20 eV). For the graphene-supported 20S proteasome, 50 micrographs with a pixel size of 1.0742 Å were collected via AutoEMation software (52) on a Thermo Fisher Scientific Titan Krios (300 kV) TEM equipped with a K3 camera (Gatan, Inc.) and a GIF-Quantum energy filter (20 eV). For the cryo-EM dataset of spike protein in graphene sandwich, 2,200 micrographs were automatically collected via AutoEMation software on a Thermo Fisher Scientific Titan Krios G3i (300 kV) TEM equipped with a K3 camera (Gatan, Inc.) and a GIF-Quantum energy filter (20 eV). During data collection, all these micrographs were fractionated to 32 frames with a total dose of 50 e^−^/Å^2^, and motion-corrected through MotionCor2 (5). The motion-corrected micrographs were imported into CryoSPARC (53) for patch CTF estimation, micrograph interpretation, and particle picking. The particles were then imported into Relion (54) for several rounds of 2D and 3D classification and the final 3D reconstruction. The particle numbers used for the final 3D reconstruction of graphene-sandwiched and graphene-supported apoferritin, graphene-sandwiched, and graphene-supported 20S proteasome were 9,596, 7,977, 14,392, and 48,637, respectively, and the reported resolutions were 3.1 Å, 3.7 Å, 2.6 Å, and 2.4 Å, respectively. For cryo-EM reconstruction of spike protein, we picked 1,181,810 particles from the raw micrographs, and 418,743 particles were kept for the final 3D reconstruction (*SI Appendix*, Table S1). PDB 6PXM (https://www1.rcsb.org/structure/6PXM), 3J9I (55), and 8HRI (https://www.rcsb.org/structure/8HRI) were used to dock into the density maps of apoferritin (EMD-37156), 20S proteasome (EMD-37158) and spike (EMD-38213) protein, and structural visualization was performed in Chimera (56).

The thickness of ice in 193 holes was estimated using a previously published method (39). In brief, we began by capturing a micrograph with an energy filter (20 eV), followed by capturing another micrograph without utilizing the energy filter. Subsequently, we determined the intensity of the micrograph taken with the energy filter (referred to as *I_zlp_*) and the micrograph without the energy filter (referred to as *I_0_*). The thickness was then calculated using the following formula:

$$d=\Lambda ln\frac{I_{0}}{I_{\mathrm{zlp}}},$$

where Λ is the apparent mean free path for inelastic scattering (380 nm) (57).

The per-frame B factor was obtained through Bayesian polishing in Relion (54) and plotted against the electron dose. The overall B factor was calculated using a previously published method (48) which relies on the reported resolution and corresponding particle number. In order to determine the beam-induced particle motion, we initially performed Bayesian polishing in Relion (54) to identify the particle coordinates in each frame. Subsequently, we calculated the root-mean-squared displacement of particle coordinates at a specific frame relative to those at the first frame for Fig. 4*C*, and the root-mean-squared displacement of particle coordinates at a specific frame relative to those at the previous frame for *SI Appendix*, Fig. S13.

### Characterization of the Charging Effect.

Micrographs were acquired using two modes: the low-magnification (340×) mode with a dose rate of 1.2 × 10^−4^ e^−^/(Å^2^ s) at the defocus of −40 mm and the high-magnification mode (3,800×) with a dose rate of 0.1 e^−^/(Å^2^ s) and an illumination area of 4.5 μm in diameter. First, a low-magnification micrograph was captured to represent the uncharged state. Then, the mode was switched to high magnification to illuminate the sample for a specific duration, ranging from 1 to 4 s. Afterward, another low-magnification micrograph was taken at the same region to measure the diameter of the charging footprint. The measured diameters were then plotted against the irradiation dose. The experiments for each irradiation dose were repeated at least three times on different squares.

### Estimation of the Effective Young’s Modulus.

Ice buckling happens when the compression in the ice layer exceeds the threshold value *N*_0_. *N*_0_ is determined by ref. 12.

$$N_{0}=A\frac{Eh^{3}}{12a^{2}\left(1-\sigma^{2}\right)},$$

where *A* is a constant dependent on the ice boundary conditions; *h* is the ice thickness; α is the radius of the ice plate; σ is the Poisson ratio; and *E* is the composite Young’s modulus of the graphene and ice. Therefore, the compression *N*_0_ in the ice is directly proportional to the composite Young’s modulus *E*, which is given by ref. 20.

$$E_{Ice-G}=E_{\mathrm{Ice}}\frac{h_{\mathrm{Ice}}}{h_{\mathrm{Ice}}+h_{G}}+E_{G\;}\frac{h_{G}}{h_{\mathrm{Ice}}+h_{G}}$$

where *h_Ice_* is the ice thickness (we used 30 nm here); *h_G_* is the graphene thickness (~0.34 nm for monolayer graphene film); *E_Ice_* is the Young’s modulus of ice, 1 GPa; and *E_G_* is the Young’s modulus of graphene, 940 GPa (46). Based on the above formula, the composite Young’s modulus *E_Ice-G_* was about 11.5 GPa for the monolayer graphene-supported sample and about 21.8 GPa for the graphene-sandwiched sample with monolayer graphene films at both sides.

### Cryo-ET Data Acquisition and Analysis.

The cryo-ET tilt series were acquired on a Thermo Fisher Scientific Titan Krios (300 kV) equipped with an energy filter and a Gatan K3 detector using SerialEM software (58), with tilting angles ranging from +60 to −60° at 3° intervals. The imaging defocus was set to −5 μm, with a pixel size of 1.358 Å, and each tilt was fractionated to eight frames, with a total dose of 2 e^−^/Å^2^. The beam-induced motion was corrected using MotionCor2 (5), and the tilt series were subsequently imported into IMOD (59) for alignment and tomogram reconstruction.

## Supplementary Material

Appendix 01 (PDF)

Movie S1. The process of transferring graphene occurred after the copper substrate had been etched away.

## Data Availability

The cryo-EM density maps of graphene-sandwiched apoferritin, 20S proteasome and spike have been deposited into EMDB under the accession numbers of EMD-37156 (60), EMD-37158 (61), and EMD-38213 (62). The raw cryo-EM movies and particle stacks of graphene-sandwiched apoferritin, 20S proteasome and spike have been deposited into EMPIAR under the accession numbers of EMPIAR-11649 (63), EMPIAR-11651 (64), and EMPIAR-11810 (65).
