# Supplementary material for: Graphene sandwich–based biological specimen preparation for cryo-EM analysis
Source: Proc Natl Acad Sci U S A. 2024 Jan 22;121(5):e2309384121. doi: 10.1073/pnas.2309384121 (PMC10835136; doi:10.1073/pnas.2309384121)
Supplement: Supplementary file 1 — Appendix 01 (PDF) [file pnas.2309384121.sapp.pdf]

PNAS

Supplementary Information for

Graphene sandwich-based biological specimen preparation for cryo-EM analysis

Jie Xu[1,2,7], Xiaoyin Gao[3,7], Liming Zheng[3,7], Xia Jia[1,2], Kui Xu[1,2], Yuwei Ma[4], Xiaoding Wei[4], Nan Liu[1*], Hailin Peng[3,5,6*], Hong-Wei Wang[1,2*]

[1]*Ministry of Education Key Laboratory of Protein Sciences, Beijing Frontier Research Center for Biological Structure, Beijing Advanced Innovation Center for Structural Biology, School of Life Sciences, Tsinghua University, Beijing 100084, China.*

[2]*Tsinghua-Peking Center for Life Sciences, School of Life Sciences, Tsinghua University, Beijing 100084, China.*

[3]*Beijing National Laboratory for Molecular Sciences, College of Chemistry and Molecular Engineering, Peking University, Beijing 100871, China.*

[4]*State Key Laboratory for Turbulence and Complex System, Department of Mechanics and Engineering Science, College of Engineering, Peking University, Beijing 100871, China.*

[5]*Beijing Graphene Institute (BGI), Beijing 100095, China.*

[6]*Academy for Advanced Interdisciplinary Studies, Peking University, Beijing 100871, China.*

[7]These authors contributed equally to this work.

*Correspondence should be addressed to H.W., H.P., or N.L.

(e-mail: hongweiwang@tsinghua.edu.cn, hlpeng@pku.edu.cn or nanliuem@tsinghua.edu.cn)

Classifications: Biological Sciences; Biophysics and Computational Biology

Keywords: cryo-EM, graphene sandwich, charging effect, beam-induced motion

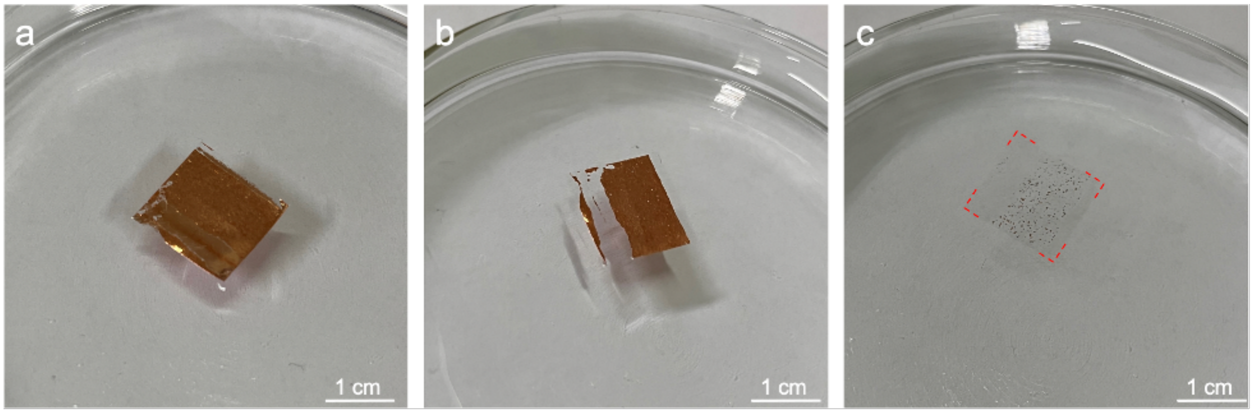

Figure S1. Production of free-standing graphene film. a, The graphene on copper foil was firstly coated by stearic acid molecules and floated on the etchant (ammonium persulfate) surface. **b-c,** The copper foil was being etched while the graphene was kept stretched (**b**) and finally free-standing on the etchant surface (**c**). The four corners of graphene are labeled by red dotted lines for clarity in (**c**).

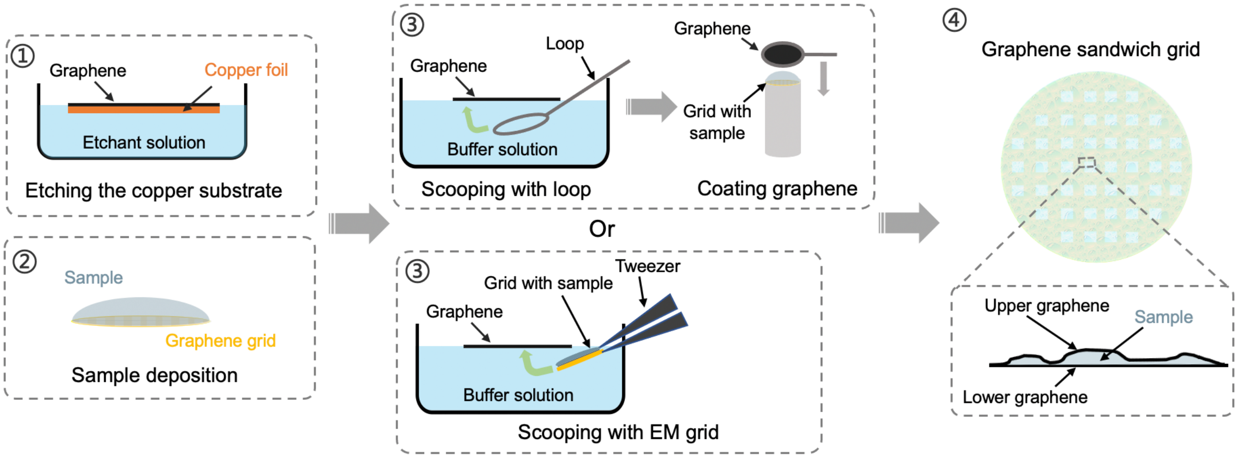

Figure S2. Diagram showing the fabrication procedure of the graphene sandwich. In the first step, the free-standing graphene on the solution surface was obtained by etching off the copper foil. In the second step, sample was deposited onto a graphene grid. In the third step, we employed two alternative methods to transfer the free-standing graphene onto the sample-loaded graphene grid, using a loop-assisted transfer method (upper) or using the graphene grid to directly scoop the free-standing graphene (lower). In the last step, the graphene sandwich grid was placed onto a piece of filter paper to enable the paper to absorb any excess solution from the sandwich area.

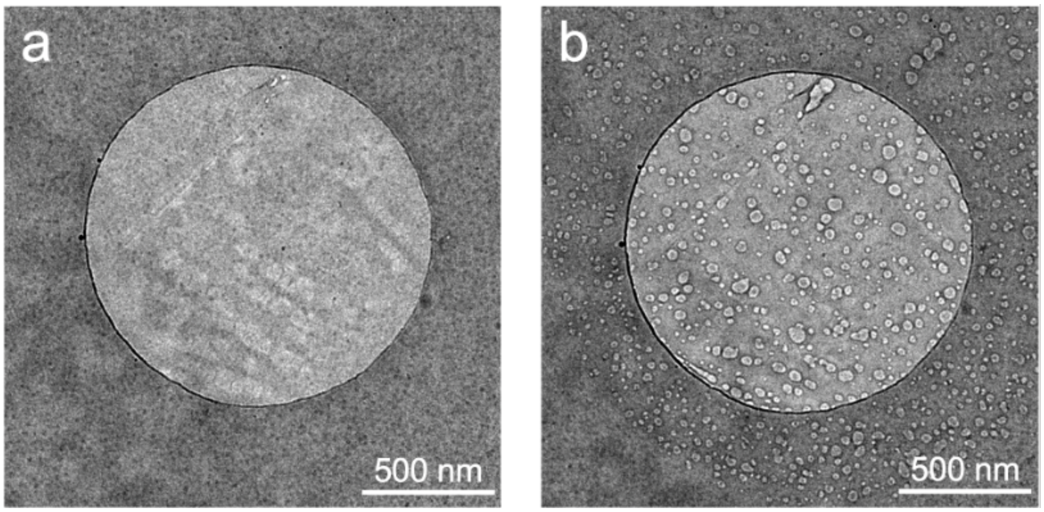

Figure S3. Room-temperature TEM characterization of graphene sandwich. a, A representative micrograph of the graphene sandwich prior to high-dose electron radiation damage. **b,** A representative micrograph of the graphene sandwich after electron radiation damage. Numerous bubbles have emerged in the irradiation area.

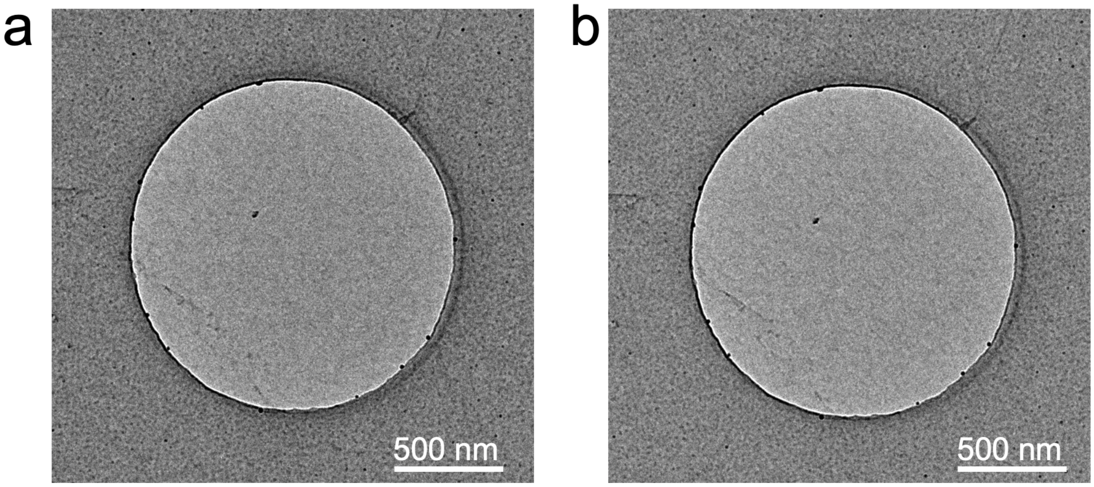

Figure S4. Micrographs of graphene with stearic acid coating. a, The image before high-dose electron radiation. **b,** The image after high-dose electron radiation.

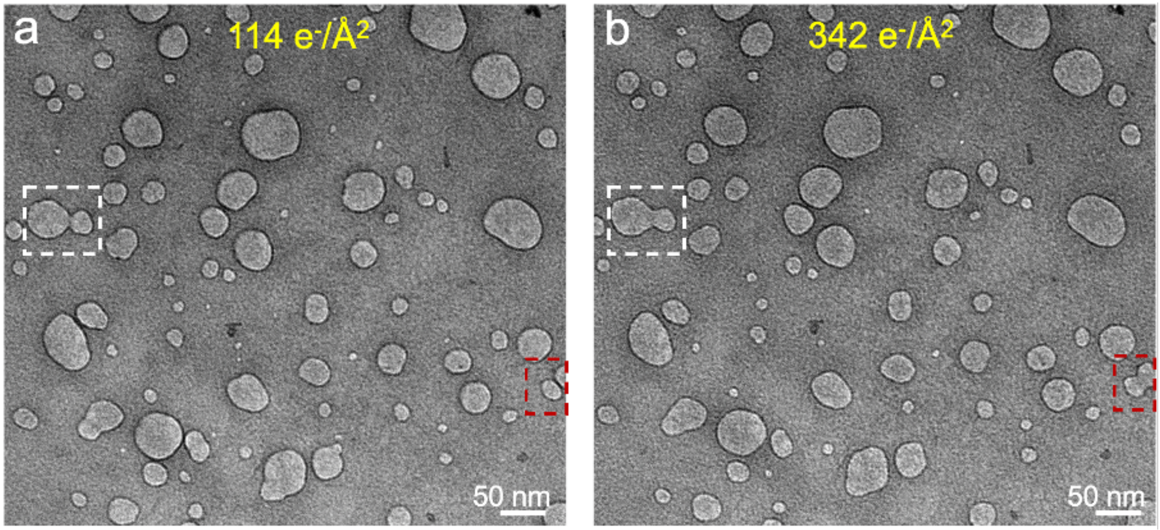

Figure S5. Room-temperature TEM characterization of graphene sandwich. a, A representative micrograph of the graphene sandwich after irradiation of 114 $e^{-}/Å^{2}$ electron dose. **b,** A representative micrograph of the graphene sandwich after 342 $e^{-}/Å^{2}$ electron dose irradiation. After longer irradiation, the majority of the bubbles increased in size. Bubble fusion events were labeled by the dotted boxes.

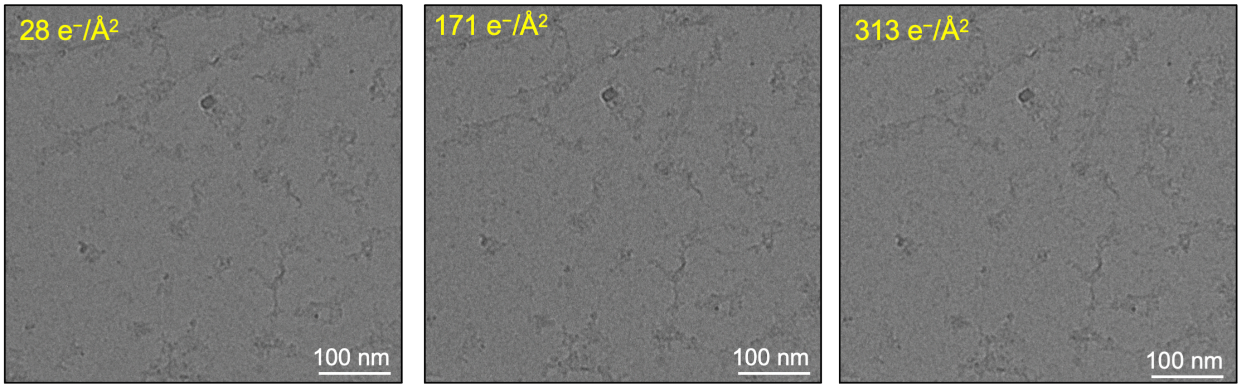

Figure S6. Imaging two stacked graphene layers at room temperature under various electron doses reveals no visible bubbles. The particles showing significant contrast in these micrographs are likely contaminants introduced during the graphene transfer process.

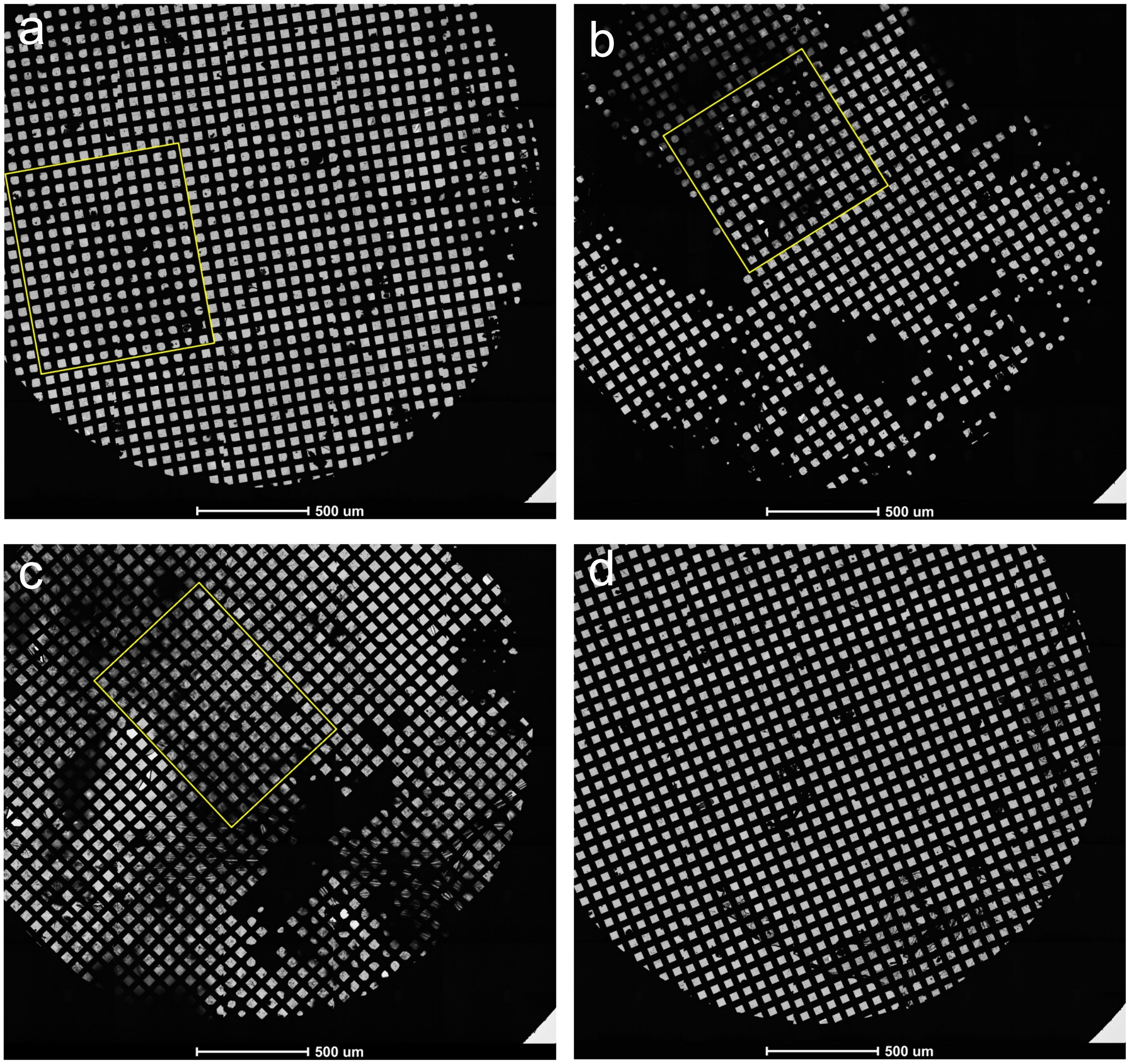

Figure S7. Representative grid atlases of graphene-sandwiched samples. **a-c**, Grid atlas of good graphene-sandwiched sample, with typical areas for data collection marked by yellow boxes. **d**, Grid atlas of not-so-good graphene-sandwiched sample, where the ice thickness was too thin.

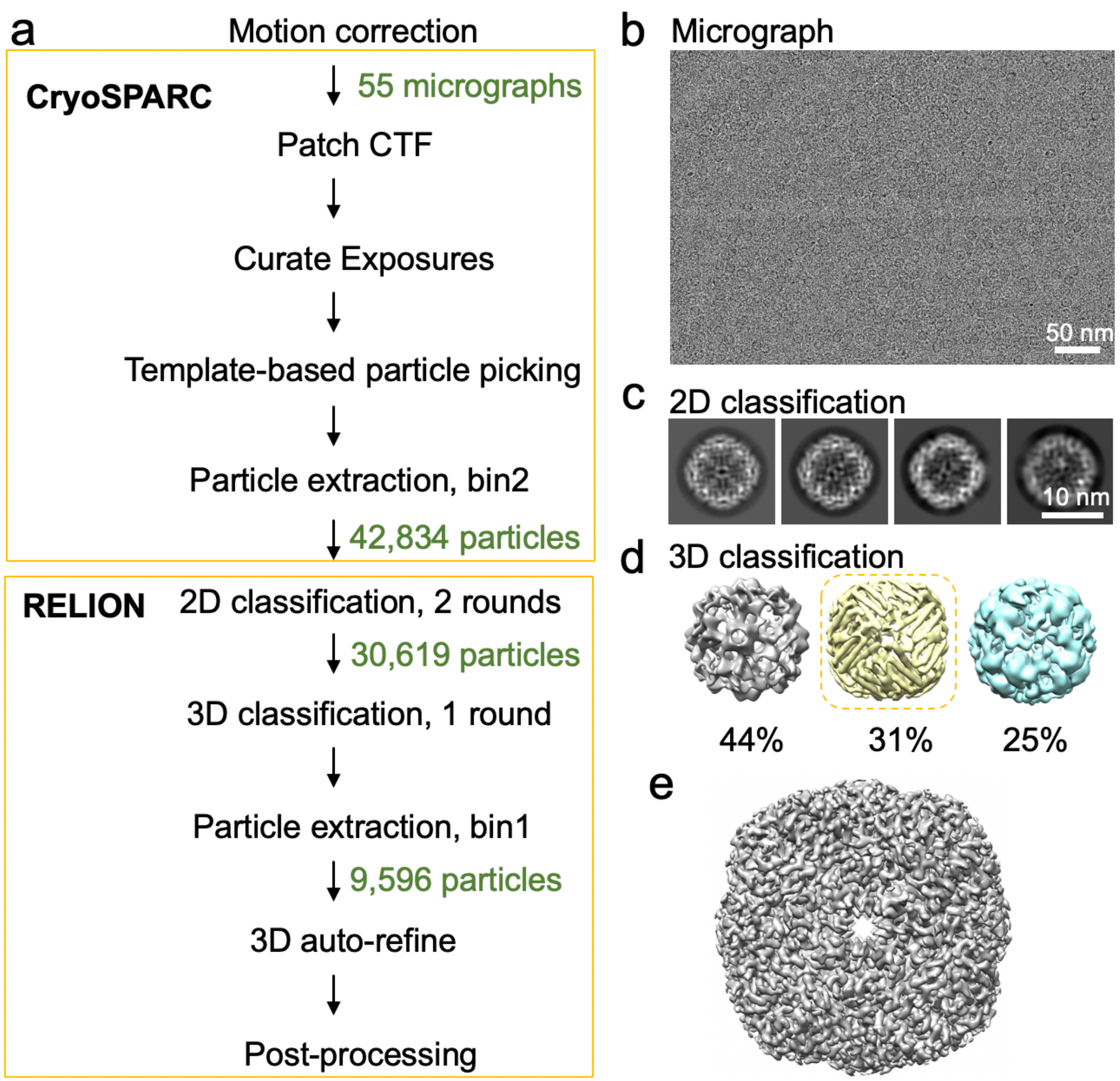

Figure S8. Workflow of the cryo-EM structure determination of apoferritin in graphene sandwich. a, The 3D reconstruction steps of apoferritin in CryoSPARC[1] and Relion[2]. **b,** A typical cryo-EM micrograph of apoferritin in graphene sandwich. **c-d,** The 2D (**c**) and 3D (**d**) classification results, respectively. **e,** The final reconstruction density map at 3.1 Å resolution.

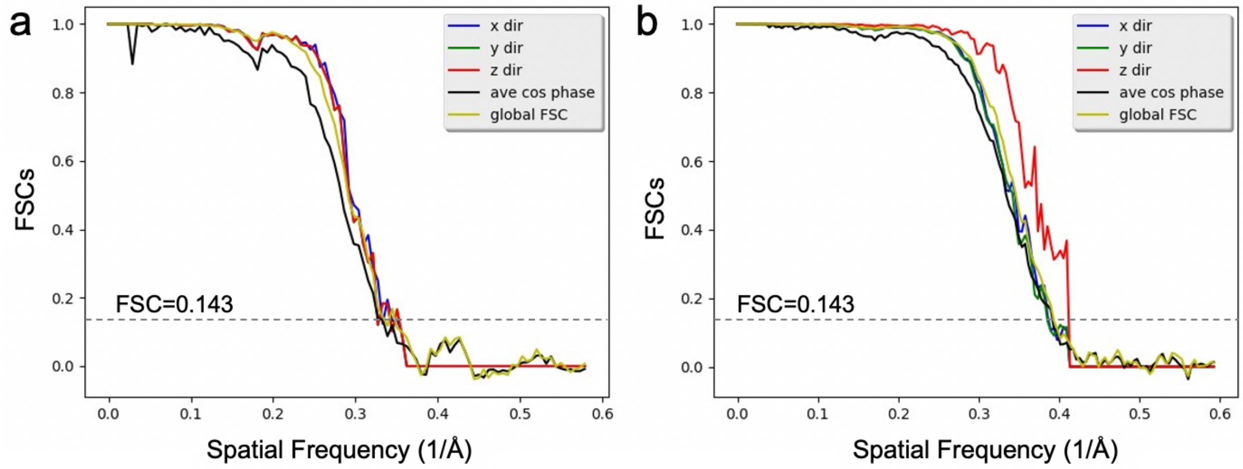

Figure S9. Directional Fourier shell correlation (FSC) profiles[3] of apoferritin (a) and 20S proteasome (b) reconstruction by using particles in graphene sandwich.

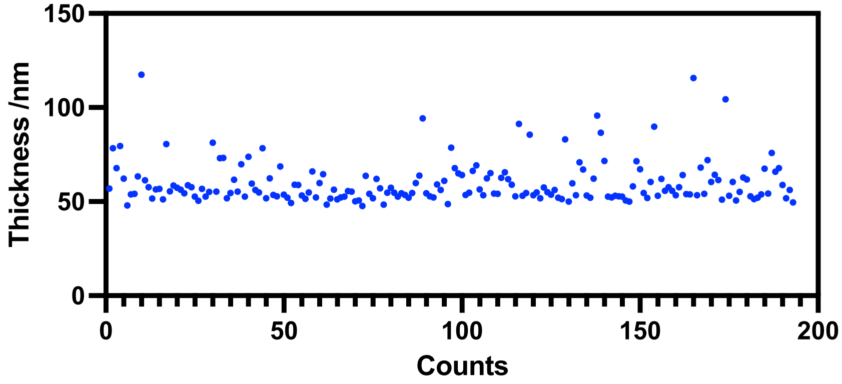

Figure S10. Thickness calculation of 193 holes on the graphene-sandwiched grid using the energy filter.

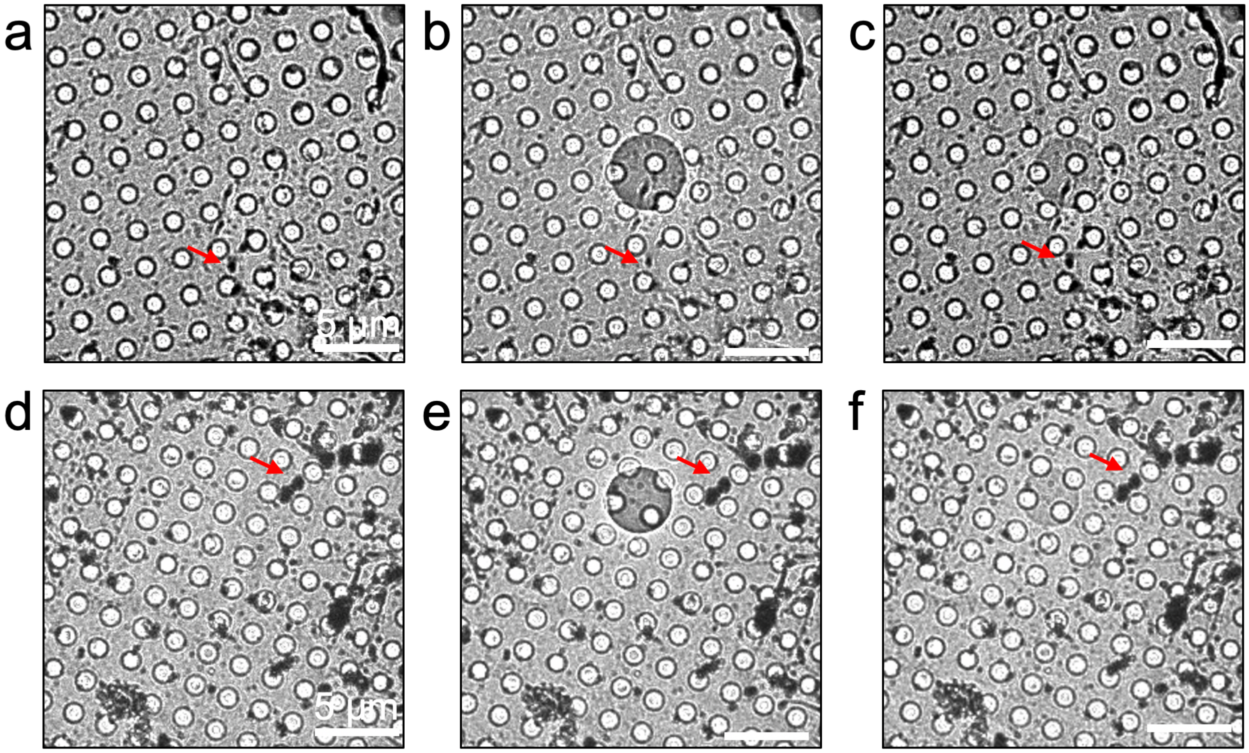

Figure S11. Characterization of charging effect on the graphene-supported ice (a-c) and the graphene-sandwiched ice (d-f). (**a**) and (**d**) were obtained prior to irradiation-induced charging. (**b**) and (**e**) were obtained immediately after irradiation with a dose of 0.3 $e^-/Å^2$ dose at a 4.5-μm diameter region, which exhibited noticeable dark footprints. (c) and (f) were prolonged irradiation of the entire field of view, under the same conditions as (**b**) and (**e**), during which the dark contrast footprints faded away. It is worth noting that the contrast of contamination particles surrounding the irradiation region in (**b**), indicated by the red arrow, became brighter compared to (**a**), and then returned to its original state as the charging dissipated in (**c**), while the contrast of contamination particles remained relatively constant in (**d-f**).

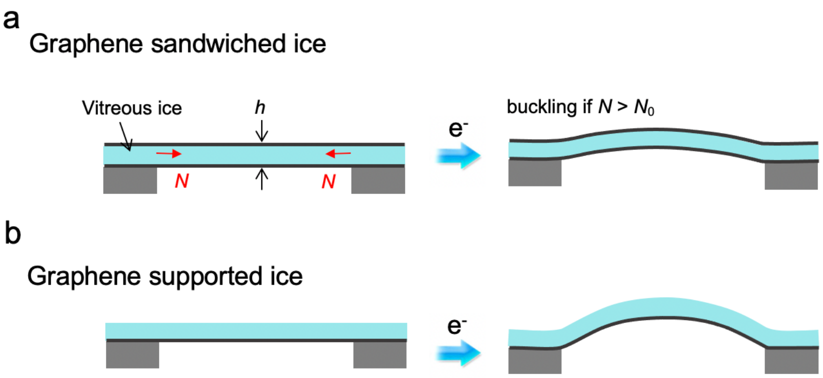

Figure S12. Diagrams showing the buckling of the graphene-sandwiched ice (a) and the graphene-supported ice (b). *N* is the compressive stress and *h* is the ice thickness in (**a**).

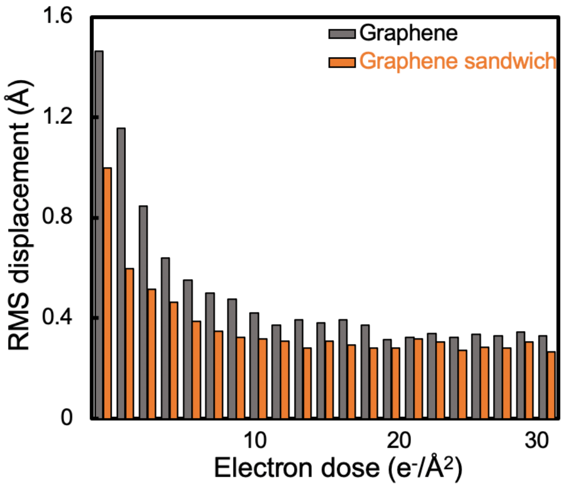

Figure S13. Particle motion with electron dose. The average particle displacement was measured for both the graphene-sandwiched sample (orange) and the graphene-supported sample (black) as a function of accumulated dose. The particle displacement was determined by measuring the distance between particles in the current frame and the previous frame. In the first 8~9 e^{-}/Å^{2}, the particle displacement on the graphene support was notably larger, approximately 1.5-2.0 times greater, compared to that observed in the graphene sandwich.

Supplementary Video 1. The process of transferring graphene occurred after the copper substrate had been etched away.

Table S1. Cryo-EM data collection, refinement, and validation statistics

	Apoferritin in graphene sandwich	Apoferritin on graphene	20S proteasome in graphene sandwich	20S proteasome on graphene	Spike in graphene sandwich
Magnification	81,000	81,000	105,000	81,000	81,000
Voltage (kV)	300	300	300	300	300
Electron exposure (e^{-}/Å^{2})	50	50	50	50	50
Micrographs (no.)	55	100	50	50	2,200
Defocus range (μm)	1.2-2.0	0.9-1.6	1.3-2.4	1.2-2.0	0.5-2.7
Pixel size (Å)	0.856	0.856	0.8374	1.0742	1.0825
Symmetry imposed	O	O	D7	D7	C3
Initial particle images (no.)	42,834	82,882	15,746	50,414	1,181,810
Final particle images (no.)	9,596	7,977	14,392	48,637	418,743
Map resolution (Å)	3.1	3.7	2.6	2.4	2.5
FSC threshold	0.143	0.143	0.143	0.143	0.143

References

1. Punjani, A., Rubinstein, J. L., Fleet, D. J. & Brubaker, M. A. cryoSPARC: algorithms for rapid unsupervised cryo-EM structure determination. Nat Methods 14, 290-296, doi:10.1038/nmeth.4169 (2017).

2. Scheres, S. H. RELION: implementation of a Bayesian approach to cryo-EM structure determination. J Struct Biol 180, 519-530, doi:10.1016/j.jsb.2012.09.006 (2012).

3. Tan, Y. Z. et al. Addressing preferred specimen orientation in single-particle cryo-EM through tilting. Nat Methods 14, 793-796, doi:10.1038/nmeth.4347 (2017).
